# Three centuries of biogeochemical change in a temperate embayment as revealed by sediment core stable isotopes, radiometric dating, and historical ecology

**DOI:** 10.3354/meps14807

**Published:** 2025-03-27

**Authors:** Sawyer J. Balint, Morgan Schwartz, Andrew Gray, Tim Cranston, Robinson W. Fulweiler, Melissa Ederington-Hagy, Rick McKinney, Autumn Oczkowski

**Affiliations:** 1 Department of Earth and Environment, Boston University, Boston, MA 02215, USA; 2 Student Volunteer, US EPA Atlantic Coastal Environmental Sciences Division, Narragansett, RI 02882, USA; 3 US EPA Atlantic Coastal Environmental Sciences Division, Narragansett, RI 02882, USA; 4 Department of Environmental Sciences, University of California Riverside, Riverside, CA 92521, USA; 5 Department of Water Supply, Town of North Kingstown, North Kingstown, RI 02852, USA; 6 Department of Biology, Boston University, Boston MA 02215, USA

**Keywords:** Sediment cores, Stable isotopes, Historical ecology, Narragansett Bay, Eutrophication

## Abstract

Efforts to improve water quality in urbanized embayments may be complicated by changes that predate contemporary ecological monitoring efforts. Such is the case in Wickford H arbor, Rhode Island, one of the oldest continuous settlements in the northeastern USA, that is exhibiting degraded water quality after centuries of land use change, physical modifications, and nutrient loading. Here, we used historical ecology and sediment geochemical records to discern the biogeochemical impacts of these anthropogenic forcings over time. Segmented linear regressions fitted to the radiometrically dated sediment cores found break points in the geochemical record that align with physical modifications in the 1800s and nutrient enrichment in the 1930s. Reductions in grain size and sorting over time suggest that railway construction in the late 1800s constrained the hydrodynamic flushing of the study system and is an important driver of current water quality. Ratios of bulk carbon, nitrogen, and phosphorus content are indicative of a system that has been persistently eutrophic. Indeed, bulk N isotope composition reflects a 5‰ increase in δ^15^N since the colonial era, representing a shift to anthropogenic N sources that accompanied regional land use change. Subsequent increases in bulk C stable isotope composition and biogenic silica concentration suggest that primary production increased during the 18^th^ and late 20^th^ centuries. This work illustrates how ecological changes contributing to poor water quality can occur prior to con temporary nutrient loading, and efforts to restore systems in the absence of a historical ecological baseline are unlikely to produce a predictable ecosystem recovery.

## 1. INTRODUCTION

Understanding the complex historical relationship between humans and their environment requires a focus on coastal systems. In many regions of the world, the human population has and continues to be disproportionally concentrated along estuaries and coastlines (Barragán & de Andrés 2015, Holmes et al. 2022). Global population growth, industrialization, and land-use change have led to immense modifications of these systems (Kummu et al. 2016, Tanaka et al. 2021), with anthropogenic nutrient inputs particularly common in urbanized areas (Bernhardt et al. 2008, Freeman et al. 2019). Subsequent eutrophication, anoxia, and changes in species composition in the late 20^th^ century have threatened valuable ecosystem services (Mehvar et al. 2018) and have led to a renewed effort to combat poor water quality, often through targeted reductions in nutrient loading (Bricker et al. 2008, Howarth 2008, Jessen et al. 2015). In many cases, managers have had success in reducing point-source nutrient loading while struggling to identify and mitigate non-point sources (Van Meter et al. 2016, Van Meter & Basu 2017). The result of nutrient mitigation efforts have been mixed (Duarte et al. 2009), with many systems failing to return to restoration targets despite large reductions in point-source nutrient loading.

The reasons for failing to meet restoration targets are complex. One major driver is that as nutrient loading changed, there were simultaneous changes across a variety of spatial and temporal scales that predated contemporary scientific monitoring efforts (McClenachan et al. 2012, López-Merino et al. 2017). In many estuaries, exploitative fishing and shellfish harvesting led to substantial ecosystem shifts in the 19^th^ century (Kirby 2004, Rick et al. 2016), physical modifications and infrastructure development altered tidal flushing (Bice et al. 2023, Phlips et al. 2023), and climate change has resulted in warming, acidification, and sea level rise (Scanes et al. 2020, Wåhlström et al. 2020, Khojasteh et al. 2021). These cumulative impacts of human development occurring over decades or centuries can produce ‘shifting baselines’ which can greatly complicate our understanding of how estuaries will respond to mediation efforts such as point-source nitrogen (N) reductions (Duarte et al. 2009, Pedretti & Robson 2022). Developing a robust understanding of how these distinct environmental forcings impact ecosystem function using an interdisciplinary approach is needed to contextualize contemporary attempts to improve water quality.

Elemental and stable isotope analyses of sediment cores are routinely employed to detect historical changes to depositional environment, nutrient cy cling, and ecology in coastal systems across the world (e.g. Vaalgamaa et al. 2013, Cerda et al. 2016, Gao et al. 2017, Castelo et al. 2021, Amora-Nogueira et al. 2023, Li et al. 2023, Muniz et al. 2024, Volante et al. 2024, Wang et al. 2024). Additionally, qualitative documentary evidence (e.g. journal entries, paintings, newspapers, maps, surveys, photographs) has been used to reconstruct human impacts on marine systems (Lotze et al. 2006, Kemp et al. 2015, Thurstan 2022). However, pairing these 2 disciplines, namely geochemistry and historical ecology, remains an under-used approach for exploring how estuarine systems have responded to human impacts in the past. Here, we aimed to develop an ecological baseline for one of the oldest continuous settlements in Rhode Island (RI), USA, using a combination of both sediment core analyses and historical documentary references.

In this study, we first compiled oral and written historical documentation to reconstruct physical and eco logical modifications to Wickford Harbor, a temperate, shallow embayment of Narragansett Bay, RI. We then collected sediment cores and used the stable isotopic composition (δ^15^N, δ^13^C), elemental composition (N, C, P), biogenic silica (BSi) concentration, radiometric dating (^137^Cs, ^210^Pb_ex_), and physical sedimentary properties (grain size, sorting) to quantify biogeochemical changes to the system over the past 3 centuries. These biogeochemical records, when paired with extensive historical documentation of the region, allows us to discern the response of Wickford Harbor to anthropogenic pressures. We hope that the characterization of this system in the past will inform contemporary nutrient mitigation efforts while simultaneously furthering our understanding of how other heavily modified embayments may respond to reductions in nutrient loading.

## 2. MATERIALS AND METHODS

### 2.1. Site description

**Fig. 1.**
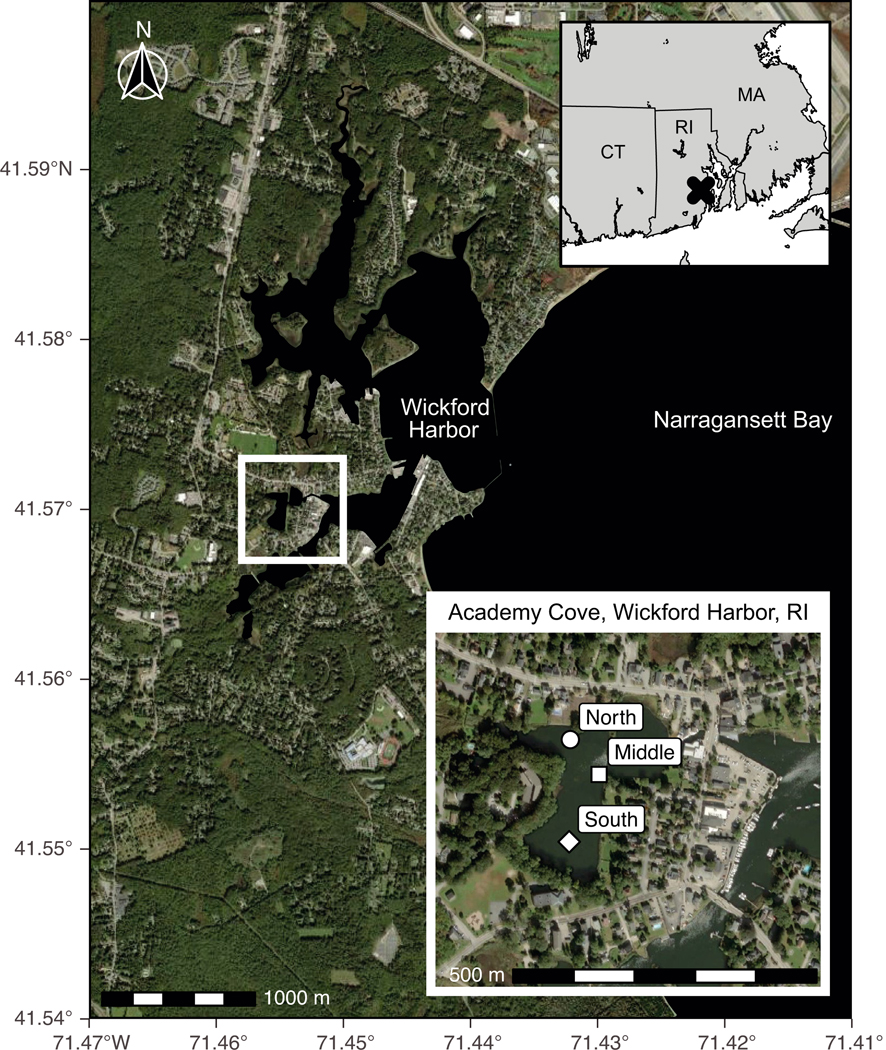
Academy Cove, Wickford Harbor, Rhode Island (USA). White symbols in Academy Cove inset indicate collection locations of sediment cores

Wickford Harbor, RI (41°34′10.13″N, 71°26′45.76″W) is an approximately 400 acre (~162 ha) complex of shallow embayments located on the southwestern coast of Narragansett Bay (Fig. 1). Here, we focus on one embayment, Academy Cove, which is surrounded by one of the oldest continuous settlements in the northeastern USA.

Oral history from Lorén Spears, enrolled Narragansett Tribal Nation citizen and Executive Director of the Tomaguag Museum (Exeter, RI), provides a glimpse into the earliest anthropogenic influences on the Wickford Harbor system prior to European settlement in the 17^th^ century. The land currently occupied by Wickford Village would have likely been used as a summer settlement by the Narragansett, where fish, seals, oysters, and saltwater plants would have been harvested (L. Spears pers. comm.). Additionally, much of the land adjacent to Academy Cove would have been used for the cultivation of corn and other crops, which were fertilized with fish. During the winter months, the Narragansett moved inland and partially survived on the food that had been collected in the summer village (L. Spears pers. comm.). This oral history is supplemented by archaeological evidence of human habitation in RI extending over 1000 years BP (Leveillee et al. 2006). The watershed around Wickford Harbor thus experienced some degree of human modification long before European contact.

Sought for its oyster fishery and natural harbor, Europeans acquired the land of Wickford in 1637 and quickly built a working port. The village of Wickford began to take its current shape in the 1700s with the construction of major roads (Cranston 2011a), and by the 1790s, the village had become an economically important port and was experiencing rapid population growth to support its agricultural, fishing, shell-fishing, and shipbuilding industries (Cranston 2011b). Major physical modifications to the system were undertaken in the early 1800s, namely the construction of bridges in 1808, 1812, and 1815 that reduced the size of the Academy Cove inlet to its p resent-day width of approximately 11 m (Cranston 2011a). The final major physical modification to the system occurred in 1899 with the construction of a railway jetty across the middle of Academy Cove. This addition, combined with construction of a rudimentary spillway in 1918, further reduced tidal flushing to the western half of the Cove.

**Fig. 2.**
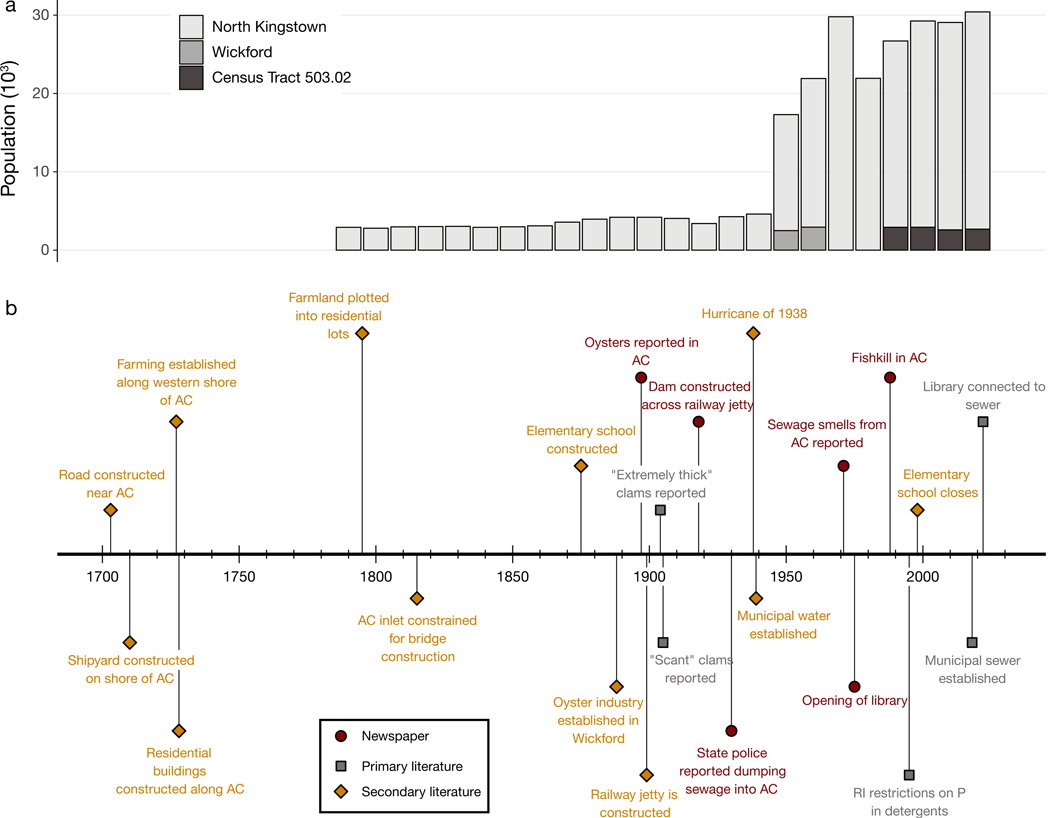
(a) Timeline of the population in Washington county and (b) notable changes to the ecology, nutrient cycling, and physical structure of Academy Cove (AC) recorded in the historical record. Wickford is an unincorporated village within the municipal borders of the Town of North Kingstown, RI, and thus the North Kingstown population encompasses the population of Wickford

Anthropogenic changes to nutrient loading into Academy Cove are also documented beginning in the 18^th^ century. Academy Cove likely received increased nutrient loading from agricultural activities from the mid-1700s onwards, and the construction of homes around the Cove in the 1820s may have induced additional sewage-derived nutrient loading (Cranston 2011a,b). However, the volume of direct sewage input would have been comparatively low until construction of a municipal water supply in 1938 (Cranston 2011a). Between the mid-20^th^ century and 2018, practically all residents and businesses relied on cesspools or septic systems to manage wastewater, resulting in groundwater-induced nutrient loading to the Wickford Harbor system. The construction of a municipal water supply in the mid-20^th^ century coincided with a large increase in the population of the town of North Kingstown, which encompasses Wickford Village (Fig. 2). However, the population within the Academy Cove watershed likely did not increase as drastically since the area had been settled over a century prior.

Primary historical documentation provides additional ecological snapshots from the past 3 centuries. Digitized copies of the local newspaper, the ‘Standard Times’ (historically called ‘The Standard’ and the ‘Wickford Standard’) noted the presence of oysters in Academy Cove in 1897, while the Thirty-Eighth Annual Report of the Rhode Island Commissioners of Inland Fisheries, published in 1908, details a transition from ‘thick clams’ in 1904 to ‘scant clams’ by 1905 (Fig. 2). A productive oyster fishery in Wickford Harbor collapsed in 1938 in response to dredging for a local military base (Cranston 2011a), and fish kills in Academy Cove were documented in the newspapers as recently as 1988.

In the present day, Wickford Harbor is heavily eutrophic. Concern over the state of the watershed (from both environmental and public health perspectives), combined with insufficient septic system capacity in the commercial district of Wickford, spurred the community to begin transitioning to a municipal sewer system in 2018. It is anticipated that this new wastewater treatment infrastructure will reduce groundwater N loading, providing a unique opportunity to observe how a historically eutrophic ecosystem will respond to this dramatic biogeochemical change.

### 2.2. Sediment core collection and analysis

Three cores were collected in December 2019 and March 2021. Cores were collected using 5.7 cm inner-diameter PVC push cores and were stored upright before they were frozen and stored at –20°C. Cores were split and sectioned in 1 cm slices from 0 to 4 cm and 2 cm slices thereafter to a maximum depth of 54 cm. Subsamples for bulk density, chronological modeling, BSi concentration, and elemental and isotopic N and C composition were dried at 60°C for at least 48 h. Bulk density samples were weighed and homogenized using a mortar and pestle for subsequent chronological modeling. Samples for elemental and isotopic analysis were homogenized with a ring and puck mill to prevent silica contamination.

**Fig. 3.**
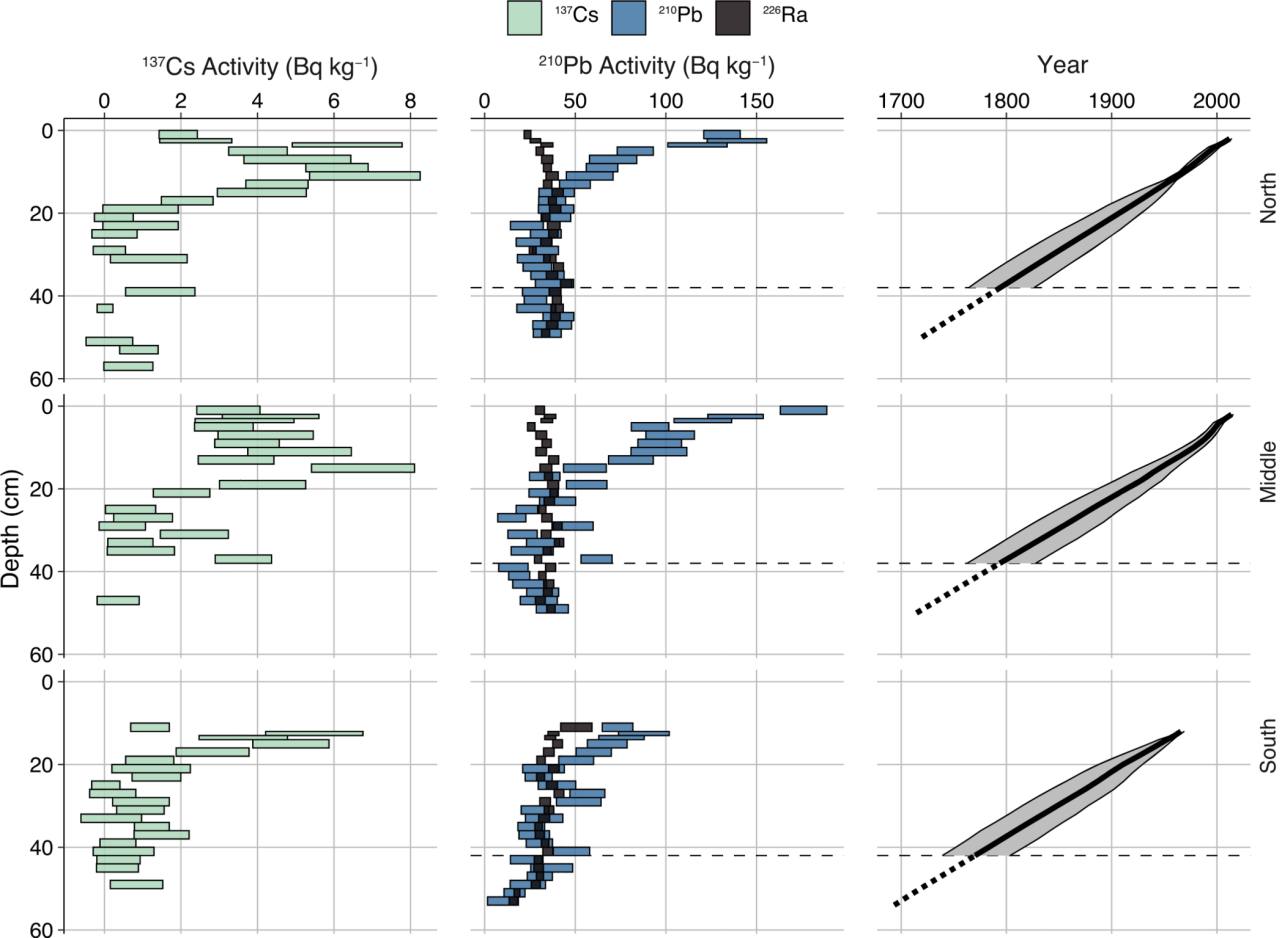
^137^Cs activity (left), ^210^Pb and ^226^Ra activity (center), and chronological models (right) of sediment cores (rows) at 3 sites in Academy Cove, RI (see Fig. 1). Horizontal rectangles indicate 1 SD, while chronological models show the 95% confidence interval. Horizontal dashed lines indicate the Markov chain Monte Carlo modeled minimum detectable ^210^Pb_ex_ activity, and the chronology below this depth is extrapolated from prior accumulation rates

Chronological models were developed for each core based on down-core abundances of ^137^Cs and excess ^210^Pb (^210^Pb_ex_). Bulk density was measured in subsamples that were then dried, homogenized, loaded into plastic vials, triple sealed, and held for at least 21 d to develop secular equilibrium between ^226^Ra and ^222^Rn. Gamma spectra were then counted on a small anode germanium well detector (Canberra GSW120 SAGe) for a minimum of 80000 s and converted to estimations of radionuclide abundance using Canberra Genie 2000 software calibrated with multi-radionuclide geometric standards that included ^210^Pb and ^137^Cs obtained from Eckert and Ziegler SE. The activity of ^210^Pb_ex_ was determined by subtracting ^214^Pb activity from total ^210^Pb activity (Fig. 3). Chronological models for each core were derived from the date of core collection (surface date), the horizon with peak ^137^Cs activity assigned as 1963 (Waters et al. 2015), and ^210^Pb_ex_ profiles using Markov chain Monte Carlo (MCMC) simulations with prior distributions for ^210^Pb influx, supported ^210^Pb, accumulation rate, and the dependence of accumulation rate on neighboring depth (Aquino-López et al. 2018). The chronology of the southern core was further adjusted to accommodate evidence of surface erosion (low ^210^Pb_ex_ activities, shallow ^137^Cs peak, and offset δ^15^N values relative to the other cores). Chronological modeling was performed using R v.4.3.3 (R Core Team 2023) with the packages ‘rplum’ and ‘rbacon’ (Blaauw & Christen 2011, Aquino-López et al. 2018).

Grain size was quantified for each core using laser diffractometry. Subsamples were gently rinsed through a 2 mm sieve using deionized water and analyzed on a Malvern Mastersizer 3000 paired with a Malvern Hydro LV automatic wet dispersion unit. Particle size distributions were analyzed for texture as described by Blott & Pye (2001) and Friedman & Sanders (1978), and the logarithmic Folk and Ward method (Folk & Ward 1957) was used to calculate mean particle size and sorting using the R package ‘G2Sd’ (Fournier et al. 2014). Bivariate plots of Mean_φ_ versus Sorting_φ_ were then used to discriminate between depositional environments over time (Lario et al. 2002, Tanner 1991, Oczkowski et al. 2020a, Watson et al. 2013).

Inorganic and total phosphorus (P) concentrations of 30 mg subsamples were determined using 10% HCl acid digestion (Strickland & Parsons 1972, Aspila et al. 1976) and spectrophotometry at 885 nm using a spectrophotometer (PerkinElmer LAMBDA 35). Additional subsamples were fumigated for 6 h with 12M HCl for organic C analysis (Harris et al. 2001). The %N, %C, δ^15^N, and δ^13^C of the fumigated and non-fumigated subsamples were quantified on a continuous flow isotope ratio mass spectrometer (Elementar VisION) interfaced with an elemental analyzer (Elementar Vario Isotope Select). Replicate analyses of the internationally recognized isotopic standard reference materials USGS40 (δ^13^C = −26.4‰, δ^15^N = −4.5‰), USGS41 (δ^13^C = +37.6‰, δ^15^N = +47.6‰), and an in-house working standard (blue mussel homogenate, δ^13^C = –18.3‰, δ^15^N = +11.2‰) were used to normalize isotopic results to air (δ^15^N) and Vienna Pee Dee belemnite (δ^13^C) scales via a multi-point linear normalization (Qi et al. 2003, Balint et al. 2024). A second working standard (NIST 1547, δ^13^C = –26.0‰, δ^15^N = +2.0‰) was used as a quality control. Measurements of USGS40, USGS41, and blue mussel homogenate yielded a pooled standard deviation (±1 σ) of ±0.6‰ for δ^13^C and ±0.5‰ for δ^15^N (n = 57). The %N and %C was calculated by comparing the peak area of the sample to a standard curve of peak area vs. elemental composition of standard reference material cystine (%C = 30.0%, %N = 11.7%) with USGS40 (%C = 40.8%, %N = 9.5%) used as a quality control. Measurements of cystine yielded a standard deviation (±1 σ) of ±0.10% for %C and ±0.10% for %N (n = 16). Isotope ratios are expressed in δ notation following the formula *X* = [(*R*_sample_/*R*_reference_) − 1], where *X* = δ^15^N when *R* = ^15^N/^14^N. Per convention, the relative difference of isotope ratios (δ) for N and C are expressed in parts per thousand (‰).

The BSi of sediment subsamples was quantified using the wet alkaline extraction technique (De-Master 1981, Conley et al. 1993). Briefly, a homogenized sample was weighed to 30 mg and digested in 1% sodium carbonate solution for 5 h, with subsamples of the digestate collected after 3, 4, and 5 h. Dissolved silica concentration of the digestate was quantified with a flow injection autoanalyzer (Seal AA3, Seal Analytical) using the molybdenum blue colorimetric method (Strickland & Parsons 1972), and BSi concentration was calculated by linear extrapolation through the 3 sample points to correct for the dissolution of mineral silicates (Schelske et al. 1986, Sauer et al. 2006). Replicate analyses of sodium hexafluorosilicate (Na_2_SiF_6_) standards for quality control yielded a standard deviation of ±0.78 μM with a method detection limit of 0.16 μM.

### 2.3. Statistical analyses

We aimed to test whether the grain size, elemental concentration, and isotopic composition of the sediment cores changed over time and, if so, whether those changes were responsive to discrete events in the historical record. First, the data from all 3 cores were combined into 1 timeseries using the chronological modeling after differences among the cores were found to be not significant (PERMANOVA, *F*_2,76_ = 1.08, p = 0.36). Segmented regression models were then fit to each variable over time, with the number of breakpoints determined by minimizing the Bayesian information criterion with a Bonferroni correction. This method identified the presence of 0, 1, or 2 breakpoints depending on the variable tested. In cases where no breakpoints were detected, the significance of change over time was tested using an unsegmented linear regression. If 1 or 2 breakpoints were identified, then the significance of those breakpoints was tested by comparing the segmented and unsegmented linear regressions with an ANOVA, which identified significant differences in all cases. Statistical tests were considered significant when p < 0.05. All statistical analyses were performed using R v.4.3.3 (R Core Team 2023), with segmented regression models fitted using the ‘segmented’ package (Muggeo 2003, 2017, Fasola et al. 2018).

The bottom 6 cm of the southern core exhibited physical and chemical differences from the remainder of the southern core and from the northern and middle cores. At depths of 40–46 cm, the %N and % C_Organic_ values were an order of magnitude higher than those of the other cores and grain size was coarser than any other sediment sample in this study. Tukey’s outlier detection was performed on the %N and %C_Organic_, confirming that these 6 cm were extreme outliers (greater than 10.1% C and 0.9% N) relative to %N and %C_Organic_ of the entire data set. The chronological model has large uncertainty at these depths, making it difficult to ensure the comparability of these subsamples to the other cores at similar depths. We have elected to present the bottom 6 cm of the southern core independently, with separate statistical analyses, so that differences over time can be better discerned in the remainder of the data set.

## 3. RESULTS

### 3.1. Sediment dating and physical composition

Chronological modeling with MCMC simulations provided consistent results for all 3 cores (Figs. S1–S3 in the Supplement at www.int-res.com/articles/suppl/m757p037_supp.pdf). The southern core showed evidence of surface erosion, and the chronological models and depths reported hereafter assume an additional 10 cm at the top of the core. All cores exhibited a logarithmic decrease in ^210^Pb_ex_ from the surface, with maximum abundances of 109 ± 17.2, 141 ± 13.9, and 51.0 ± 15.1 Bq kg^−1^ in the northern, middle, and southern cores, respectively (Table 1). The ^137^Cs maximum, corresponding to 1963, ranged from 4.87 ± 1.00 to 6.81 ± 1.44 Bq kg^−1^ and was observed at depths between 10 and 12.5 cm. Mean accretion rates ranged from 6.0 to 6.1 yr cm^–1^, and the resulting chronological models extended to the early 18^th^ century (Table 1, Fig. 3). The maximum uncertainty of the chronological models ranged from 63 to 67 yr (±95% confidence interval), with MCMC simulated limit of detections at depths between 38 and 42 cm (Table 1). In subsequent results and discussion, estimated age is only discussed for sediment depths above the modeled ^210^Pb_ex_ limit of detection.

**Fig. 4.**
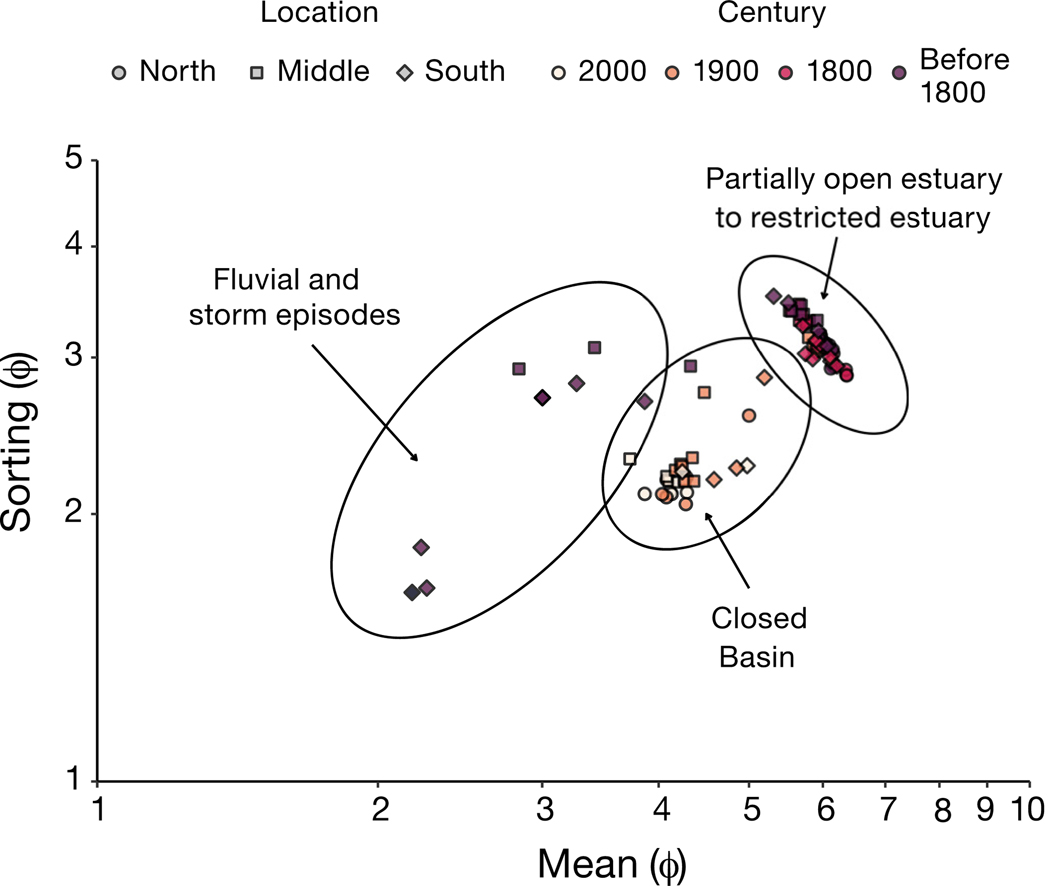
Bivariate plot of particle size mean and sorting, estimated using logarithmic Folk and Ward method. Depositional domains are modified from Lario et al. (2002) and Tanner (1991)

Granulometry revealed that the sediment was predominantly silty-sand and sandy-silt (Fig. S4). Bivariate plotting of particle size and sorting demonstrates clustering over time but not between sediment cores (Fig. 4). Samples collected prior to the 19^th^ century are almost exclusively clustered in a settling domain classified by Lario et al. (2002) as estuarine, while more recent samples cluster in a lower-energy do main associated with a closed basin. The bottom of the middle and southern cores are a notable exception, with samples exhibiting higher energy consistent with fluvial environments and storm episodes (Fig. 4).

**Fig. 5.**
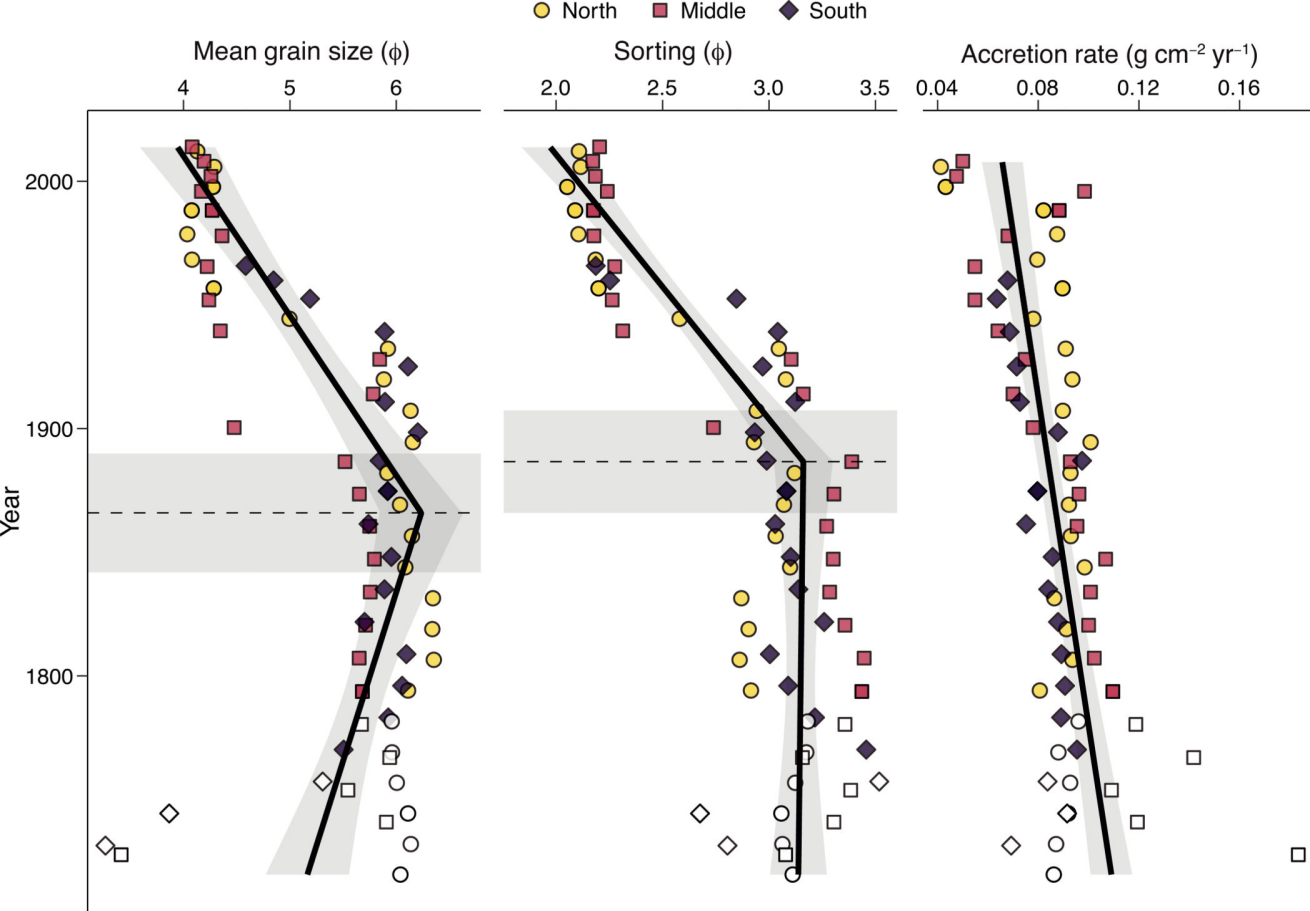
Grain size distributions and accretion rates of the sediment cores. Core locations are shown by color and shape, with samples below the minimum detectable ^210^Pb_ex_ activity indicated by lack of color. The solid black line shows the segmented linear regression, with changepoints and their associated 95% confidence intervals indicated with horizontal dashed lines and gray boxes, respectively

Sediment geochemical results are presented as the fitted values from the segmented linear regression ± the 95% confidence interval. Sediment accumulation rates (Fig. 5) exhibited a significant (p < 0.0001) linear decrease from 0.11 ± 0.009 g cm^−2^ yr^−1^ at the bottom of the cores to 0.071 ± 0.008 g cm^−2^ yr^−1^ at the surface. Conversely, grain sorting was constant from the beginning of the core until 1887 ± 21, after which it coarsened from 3.16 ± 0.14 φ to 1.97 ± 0.14 φ at the surface. Mean grain size exhibited a similar trend, with the finest grain size of 6.23 ± 0.38 φ observed in 1866 ± 24 followed by a decrease to 3.94 ± 0.35 φ at the surface.

### 3.2. Sediment nutrients and stable isotopic composition

Significant changes in the %C (p < 0.0001), %N (p < 0.0001), and %P (p = 0.014) are evident over time (Fig. S5). The %C exhibited considerable variability at depth but reached a relative minimum of 2.71 ± 0.50% in 1856 ± 26, before increasing 2-fold to 5.46 ± 0.45% at the surface. Similarly, the minimum of %N of 0.23 ± 0.03 was measured in 1894 ± 15 before increasing to 0.52 ± 0.03% at the surface. In contrast, %P increased from 1.39 ± 0.22% at the bottom of the cores to 2.67 ± 0.33 in 1950 ± 40 and remained relatively constant through the upper core.

**Fig. 6.**
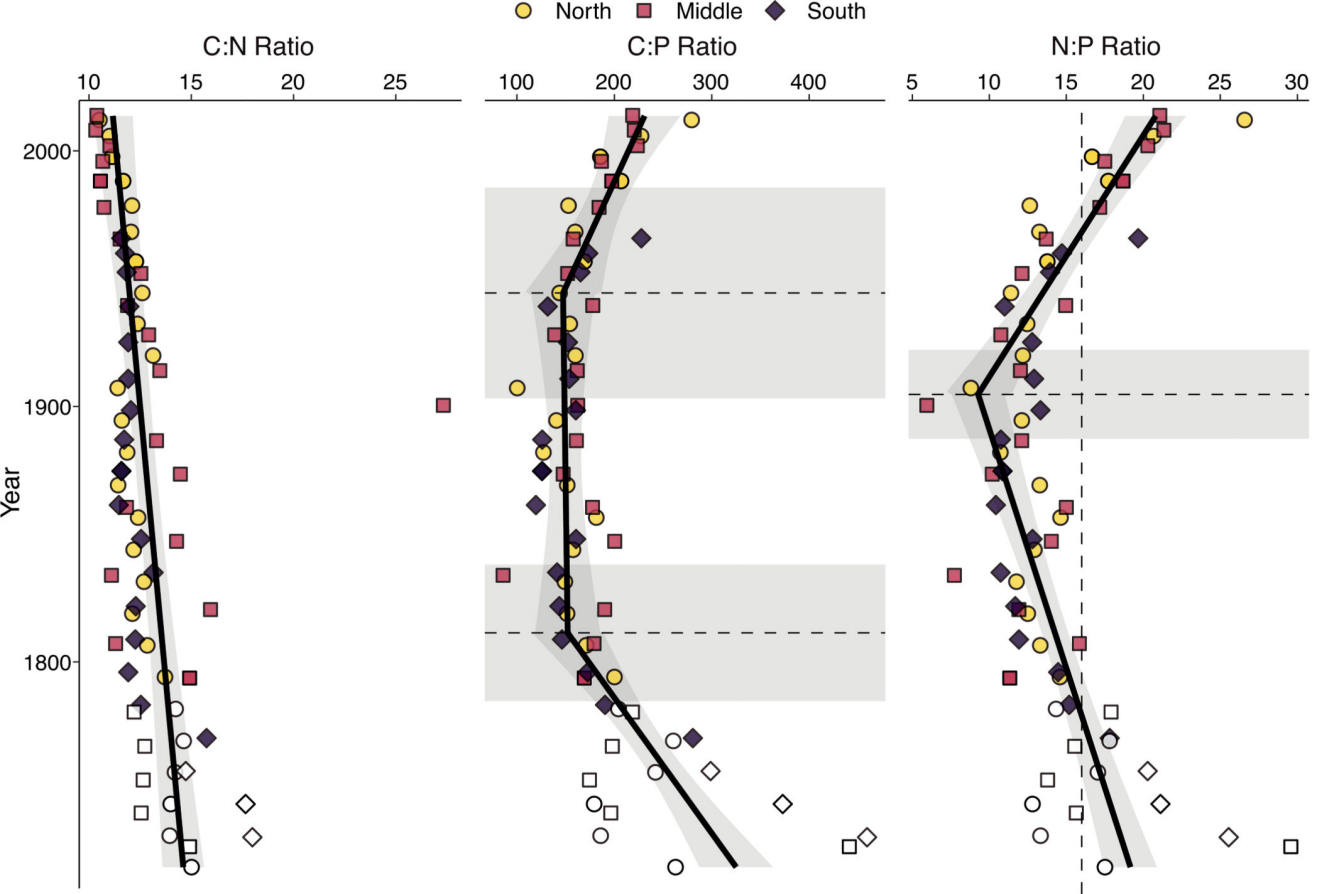
Elemental composition of sediment cores. Core locations are shown by color and shape, with samples below the minimum detectable ^210^Pb_ex_ activity indicated by lack of color. The solid black line shows the segmented linear regression, with changepoints and their associated 95% confidence intervals indicated with horizontal dashed lines and gray boxes, respectively. Vertical dashed line indicates an N:P ratio of 16:1

The ratios between these elements also exhibited differences over time (Fig. 6). The C:N ratios significantly decreased over time (p < 0.0001) from 14.7 ± 1.0 at the bottom of the core to 11.0 ± 0.9 at the surface. The ratios of C:P and N:P were more variable with time, with both ratios exhibiting significant breakpoints (p < 0.0001). The C:P ratio decreased from 326 ± 36 at the bottom of the core to 153 ± 34 in 1809 ± 26, where it remained relatively consistent until 1937 ± 43. After this breakpoint, the C:P ratio increased to present-day values of 231 ± 32. Finally, the N:P ratio decreased from 19.2 ± 1.7 at the bottom of the core to 9.2 ± 1.7 in 1905 ± 16, after which it sharply increased to 21.6 ± 1.8 at the surface.

**Fig. 7.**
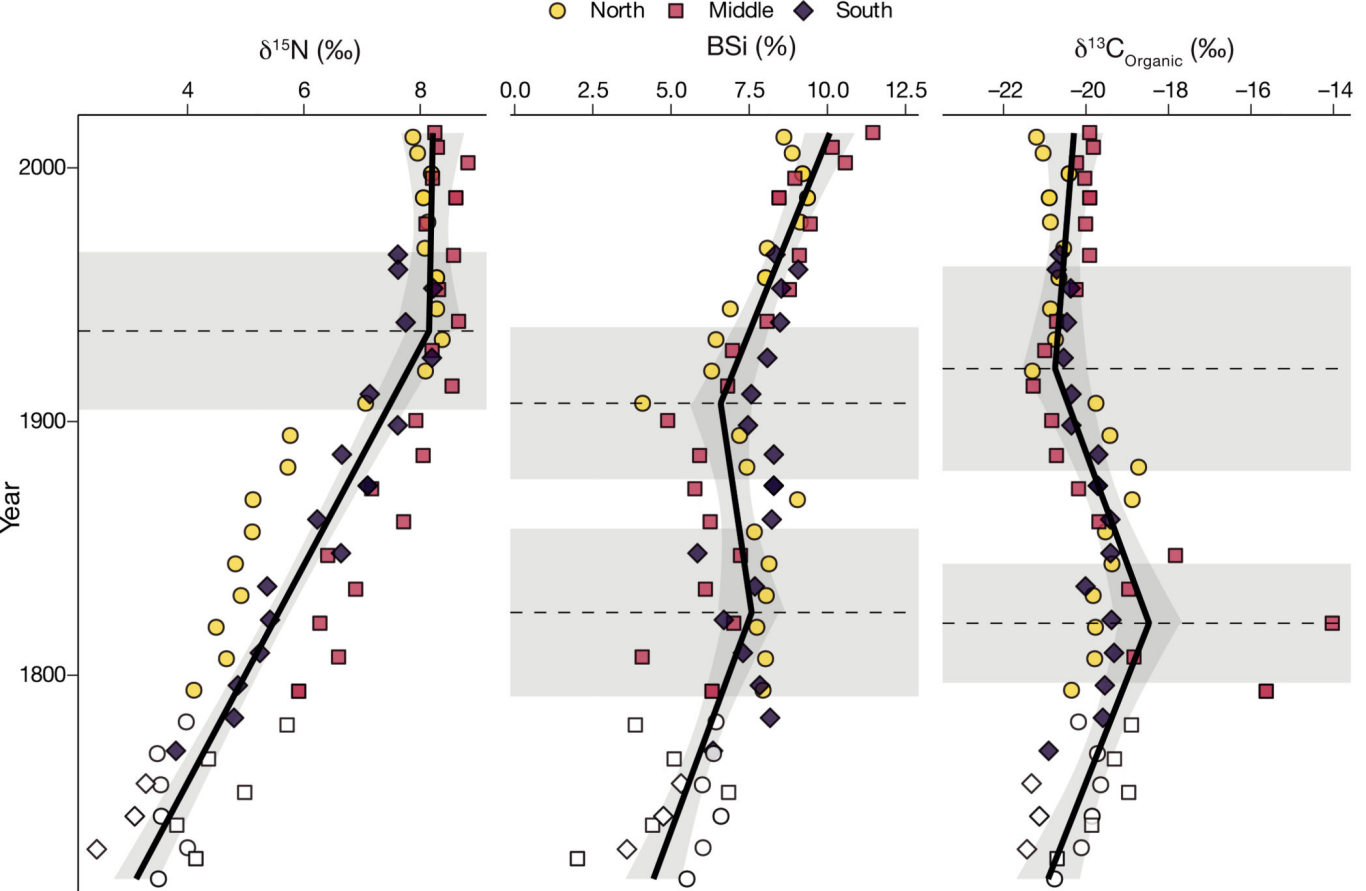
Isotopic N (left) and C (right) composition and biogenic silica (BSi) concentration (center) of sediment cores. Core locations are shown by color and shape, with samples below the minimum detectable ^210^Pb_ex_ activity indicated by lack of color. The solid black line shows the segmented linear regression, with changepoints and their associated 95% confidence intervals indicated with horizontal dashed lines and gray boxes, respectively

The %BSi (Fig. 7) exhibited 2 significant breakpoints (p < 0.0001), increasing from 4.46 ± 0.89% at the bottom of the cores to 7.61 ± 1.07% in 1825 ± 32. The %BSi then decreased to 6.51 ± 0.88% in 1906 ± 27 before increasing to 10.4 ± 0.74 at the surface. Stable isotopes of C also exhibited 2 breakpoints (p < 0.0001), with δ^13^C increasing from −20.9 ± 0.7‰ to −18.5 ± 0.8‰ in 1821 ± 23, before decreasing to –20.8 ± 0.8‰ in 1928 ± 37 and remaining relatively constant thereafter. Changes in δ^15^N (p < 0.0001) were marked by an increase from 3.1 ± 0.4‰ at the bottom of the cores to 8.1 ± 0.4 in 1933 ± 31 and remaining relatively constant through the upper core (Fig. 7).

## 4. DISCUSSION

In this study, we used historical ecology and sediment cores to investigate the biogeochemical imp acts of human development over time on a small temperate embayment. Extensive historical documentation of anthropogenic activities in this system makes Academy Cove especially well-suited to investigating the relationships between 3 centuries of human activity, nutrient loading, water quality, and ecology. The net results of this study suggest that changes to nutrient cycling in the early 1900s led to pronounced ecological effects that have the potential to be mitigated by contemporary non-point source nutrient reduction efforts, but physical and biogeochemical changes to the system that occurred over 200 yr ago also contribute to the current state of water quality in this system.

### 4.1. Ecological response to anthropogenic forcings seen through the historical record

The extensive oral history and primary and secondary historical documentation in this system allows us to reconstruct ecological changes to Academy Cove that could not be discerned with the sediment cores alone. Past work in Chesapeake Bay found evidence of sustainable oyster harvesting by Native American communities during the ~1800 yr that they inhabited the region (Rick et al. 2016), and we also suspect that human use of the ecosystem was performed sustainably until European settlement. Indeed, the 1897 ‘Standard Times’ newspaper account of oysters in the Cove paints a picture of a well-functioning benthic community that persisted at least until the 20^th^ century, but only ‘scant’ clams were observed in Aca demy Cove by 1905 (Fig. 2). Changes to watershed physical structure and declines in water quality have been identified as the drivers of oyster population collapse in larger systems (Diggles 2013), and thus the profound ecological changes that occurred in those 8 yr are likely indicative of a rapid degradation of water quality. The construction of the railway jetty in 1899 may have been a large contributor, and subsequent physical modifications to the inlet and increases to nutrient loading have only led to additional stress. Understanding the drivers of ecosystem shifts are important in historically urbanized systems because they often predate the sewage-derived nutrient loading that contemporary water quality mitigation efforts aim to address.

### 4.2. Sediment grain size and physical modifications

Changes to sediment grain size (Fig. S4) and sorting (Figs. 4 & 5) reinforce the impact of physical modifications that occurred in New England systems as they were settled in the early 18^th^ century. The historical record suggests that tidal flushing capacity in Aca demy Cove would have been drastically reduced when the inlet to the Cove was constrained and channelized in the early 1800s. Indeed, grain size statistics show a transition from a high-energy settling domain to an estuarine environment around the beginning of the 19^th^ century (Fig. 4). The construction of the railway jetty in 1899, with the addition of a rudimentary spillway in 1918, would have further constrained the western portion of the Cove and likely contributed to the transition to a lower energy depositional environment (Fig. 4) after that time. These findings are broadly consistent with past work in larger systems along the Canadian Atlantic coast, which also found that changes to hydrodynamics from bridge construction had substantial ecosystem effects (Karmakar et al. 2019, Patoine et al. 2022).

Sediment accretion rates are frequently related to land use change, particularly in freshwater lake settings. Increasing accretion rates have been broadly associated with reductions in forest cover (Schiefer et al. 2013) and increasing agricultural and population density (Baud et al. 2021). Our sediment accretion rates exhibit a moderate linear decrease over the span of our sediment cores, absent of any breakpoints that could be related to discrete events in the historical record. Documentary evidence suggests that the land within the watershed was cleared for agriculture well before 1800, and this work shows that subsequent land use change, namely the conversion of agricultural land to residential homes, did not produce a sharp response in sediment accretion rates. Indeed, the history of land use change around Academy Cove mirrors that of much of the eastern USA, which had been largely cleared for agriculture by the early 19^th^ century (Foster et al. 2008).

The bottom 6 cm of the southern core exhibited extremely high %N and %C, as well as anomalously high grain size (Table 2). Historical maps of the region point to the existence of a salt marsh in the southern portion of Academy Cove, but a nutrient-enriched salt marsh would be expected to exhibit BSi concentrations of at least 4–6% (Carey & Fulweiler 2013), rather than the 1.7% observed in our study. As previously described, this portion of the core falls well below the minimum detectable ^210^Pb_ex_ activity (Fig. 3), so it is difficult to assign a date to these data with any degree of confidence. Future work in this system could employ carbon dating to better constrain the age of this anomalous sedimentary layer, allowing for more informed speculation as to its origin.

### 4.3. δ^13^C and BSi point to increases in primary productivity over time

Long-term changes to C sources, including changes in primary production, are frequently discerned through changes in δ^13^C (Leng & Lewis 2017). The δ^13^C_organic_ is potentially reflective of the mixing of marine and terrestrial particulate organic C (Hunt 1970), dissolved CO_2_ limitation due to increased primary productivity (Fogel et al. 1992, Oczkowski et al. 2018), changes in salt marsh and seagrass composition (Serrano et al. 2016, López-Merino et al. 2017), and sediment diagenesis after deposition (Cornwell et al. 1996). Terrestrial particulate organic matter has a lower δ^13^C_organic_ than marine (i.e. algae) sources (~ −26 and −20‰, respectively, Hunt 1970), and thus the δ^13^C_organic_ values observed in these cores (~ −20‰, Table 2) suggest that terrestrial C inputs are minimal compared to marine and estuarine sources. The most defining feature of the δ^13^C_organic_ data set is the substantially enriched values recorded in the early 1800s, which are over 2‰ higher than the remainder of the core (Fig. 5). Because salt marshes and seagrass are associated with heavier δ^13^C_organic_ composition, these results may point to the existence of seagrass in the Cove at that time. A second possibility, supported by the BSi results (below), is that primary production increased in the Cove during the colonial era. The effects of organic matter degradation must also be considered, as anoxic diagenesis alters the δ^13^C_organic_ by up to −1.7‰ (Boehme et al. 1996, Lehmann et al. 2002). However, the small variability in δ^13^C_organic_ during the last century and the correspondence between δ^13^C_organic_ and BSi (Fig. 7), suggests that diagenesis is not a major driver of observed changes in δ^13^C_organic_ over time.

The BSi analyzed in the sediment cores provides an additional lens through which to view changes in primary production. BSi content can be used as a proxy for the accumulation of siliceous diatoms (Conley et al. 1993), and thus serves as a useful indicator of water column production over time (Bernárdez et al. 2005, Nazneen & Raju 2017, Li et al. 2021). The increase in BSi from the bottom of the cores to ~1825 (Fig. 5) is indicative of water column primary production increasing during the 18^th^ century, and a similar increase in BSi from ~1905 to the present day again suggests increasing production. When paired with the C isotopic record, the BSi results suggest that eutrophication in this system began at least a century earlier than the sewage-derived nutrient pollution of the 20^th^ century. These results provide additional evidence that early morphological and land use modifications may prevent the achievement of management goals.

### 4.4. Changes to N cycling as observed by δ^15^N and C:N:P ratios

With the context of the BSi and δ^13^C_organic_ results, the addition of bulk δ^15^N and elemental C, N, and P composition allow us to directly assess the relationship between eutrophication and nutrient pollution in historically urbanized systems such as Academy Cove. The δ^15^N values of sediments are reflective of N sources and N cycling within the water column and sediment, while C:N:P ratios can be used to discern their relative magnitude (Bratton et al. 2003, Vaalgamaa et al. 2013, Dang et al. 2018, Oczkowski et al. 2020b, Champlin et al. 2023). Past work in Narragansett Bay has shown natural sources of N (e.g. atmospheric N deposition, N fixation) have δ^1 5^N compositions near 0‰, while sources composed of nontreated wastewater have δ^15^N signatures greater than 7‰ (DiMilla et al. 2011, Schmidt et al. 2016, Balint et al. 2022). Thus, an increase in δ^15^N would be expected as the human population within the watershed grew and anthropogenic N inputs increased. However, increased δ^15^N can also be an indicator of eutrophication and oxygen depletion through the assimilation of enriched inorganic N generated through denitrification and nitrification (Montoya 1994, Brandes & Devol 1997) as well as the production of enriched particulate N due to fractionation during bacterial breakdown of organic N (Fogel & Cifuentes 1993). Eutrophication and subsequent oxygen depletion can result in isotopic enrichment because these conditions facilitate denitrification in both the sediments and the water column. Diagenesis further complicates interpretation of δ^15^N, with anoxic diagenesis resulting in a consistent depletion of approximately –3‰ (Lehmann et al. 2002).

The δ^15^N of sediment collected in this study exhibited a large range, from approximately 3‰ in the colonial period to over 8‰ by the 1930s, and remained relatively constant thereafter (Fig. 7). This early increase in δ^15^N corresponds to the residential settling of the watershed by Europeans, suggesting that the combination of increasing anthropogenic influence as well as eutrophication and anoxia-induced changes in N cycling during this time are reflected in the N isotopic record. Interestingly, the δ^15^N of the sediment cores had already reached present-day values prior to the construction of a municipal water supply in 1938, suggesting that anthropogenic N had already become the predominant N source by this time and/or physical modifications to the Cove reduced tidal flushing, contributing to anoxic conditions and associated denitrification. Thus, subsequent increases in groundwater N loading from wastewater in the 20^th^ century did not meaningfully change δ^15^N values in the sediment.

In contrast, the sharp increase in the N:P ratio in ~1905 aligns with increases in groundwater N loading via sewage contamination (Schelske et al. 1986, Emeis et al. 2000, Nazneen & Raju 2017, Dang et al. 2018, Copetti et al. 2019). Although the phase-out of phosphate detergents may be contributing to the observed increase in the N:P ratio in later years, the simultaneous decrease in the C:N ratio provides further evidence of a system experiencing increasing N loading (Fig. 6). Indeed, the BSi and δ^13^C_organic_ suggest increasing productivity in the 20^th^ century, which could be attributed to groundwater nutrient loading.

C:N:P ratios can also be indicative of the influence of marine and terrestrial organic matter and diagenesis, with algal biomass and phytoplankton productivity associated with a low C:N ratio (Leng & Lewis 2017, Nazneen & Raju 2017, Wigand et al. 2021, Dominik et al. 2023, Farias et al. 2024). Changes in the depositional environment also have the potential to impact C:N ratios due to the retention of terrestrial organic matter, as would differential down-core diagenesis of C, N, and P (Lehmann et al. 2002, Bratton et al. 2003). This study documented a significant linear decrease in the C:N ratio over time, but it is difficult to conclude to what extent this relationship is due to increased algae and plankton abundance, increased N concentration in the Cove due to N loading, and diageneses (Emeis et al. 2000). However, given the large magnitude of the biogeochemical forcings on this system over the past 300 yr, changes in nutrient loading are undoubtably a key constituent of the C:N:P observations.

### 4.5. Ecosystem recovery in the absence of an ecological baseline

Contemporary efforts to improve the water quality in Wickford Harbor mirror attempts in other heavily modified systems, with a focus on reducing nutrient loading (in this case, from residential and commercial sewage). In this system, successful reductions in N loading would (at best) return nutrient inputs to ~1930 levels but would not address the anthropogenic forcings that initiated eutrophication over a century prior. Legacy nutrients, ecosystem hysteresis, warming water temperature, changes to precipitation, and ongoing land use change further complicate ecosystem recovery.

This work shows that such approaches are unlikely to bring back the ecosystem functions that were once present in urbanized systems, particularly those that experienced substantial land use modification centuries ago. Although every coastal system has a unique history, this work illustrates how the ecological changes contributing to poor water quality can occur prior to contemporary nutrient loading. Indeed, we find that considerable ecological change in Academy Cove occurred prior to the introduction of municipal fresh water, and we attribute much of this change to modification of the physical structure of the Cove that occurred in the early 19^th^ century. Thus, reductions in sewage pollution provide logistical and public health benefits to the local community but do not address the foundational anthropogenic forcings on this system. The story of Academy Cove offers a lesson for water quality managers and stakeholders elsewhere: efforts to restore systems in the absence of a historical ecological baseline are un likely to produce a predictable ecosystem recovery.

## Supplementary Material

SI

## Figures and Tables

**Table 1.** Radiometric dating results for the 3 sediment cores

Location	^210^Pb_ex_ maximum	^137^Cs maximum	^137^Cs maximum	^210^Pb_ex_ limit of	Accretion	Maximum uncertainty
	(Bq kg^−1^ ± 1σ)	(Bq kg^−1^ ± 1σ)	depth (cm)	detection (cm)	rate (yr cm^−1^)	(yr, ±95% CI)

North	109 ± 17.2	6.81 ± 1.44	12	38	6.0	±63
Middle	141 ± 13.9	6.78 ± 1.35	16	38	6.1	±67
South^a^	51.0 ± 15.1	4.87 ± 1.00	12.5	42	6.1	±66

a Chronological model for the southern core assumes 10 cm of surface erosion

**Table 2.** Mean elemental and isotopic composition of the sediment cores by location. The bottom 6 cm of the southern core (outlier) is presented independently. BSi: biogenic silica

Location	Mean grain (φ)	Grain sorting (φ)	Accretion rate (g cm^−2^ yr^−1^)	%C_organic_	%N	%P_total_	%BSi	C:N	N:P	C:P	δ^15^N (‰)	δ^13^C (‰)

North	5.44	2.74	0.0859	4.03	0.331	2.23	7.43	12.5	14.9	185	6.06	−20.2
Middle	4.94	2.84	0.0927	3.89	0.316	2.1	6.76	13.1	15.8	198	7.11	−19.7
South	5.41	2.92	0.0816	4.14	0.32	2.23	7.52	12.9	14.8	195	6.16	−20.3
Outlier	2.53	2.1	0.0358	23.5	1.11	0.944	1.65	21.8	143	2980	1.05	−22.3

## Data Availability

The data and code to compute the figures and statistical testing are available in Zenodo at https://doi.org/10.5281/zenodo.10552775.
