# Supplementary material for: Three centuries of biogeochemical change in a temperate embayment as revealed by sediment core stable isotopes, radiometric dating, and historical ecology
Source: Mar Ecol Prog Ser. Author manuscript; Available in PMC 2026 Mar 27. (PMC12181943; doi:10.3354/meps14807)
Supplement: SI [file NIHMS2069992-supplement-SI.pdf]

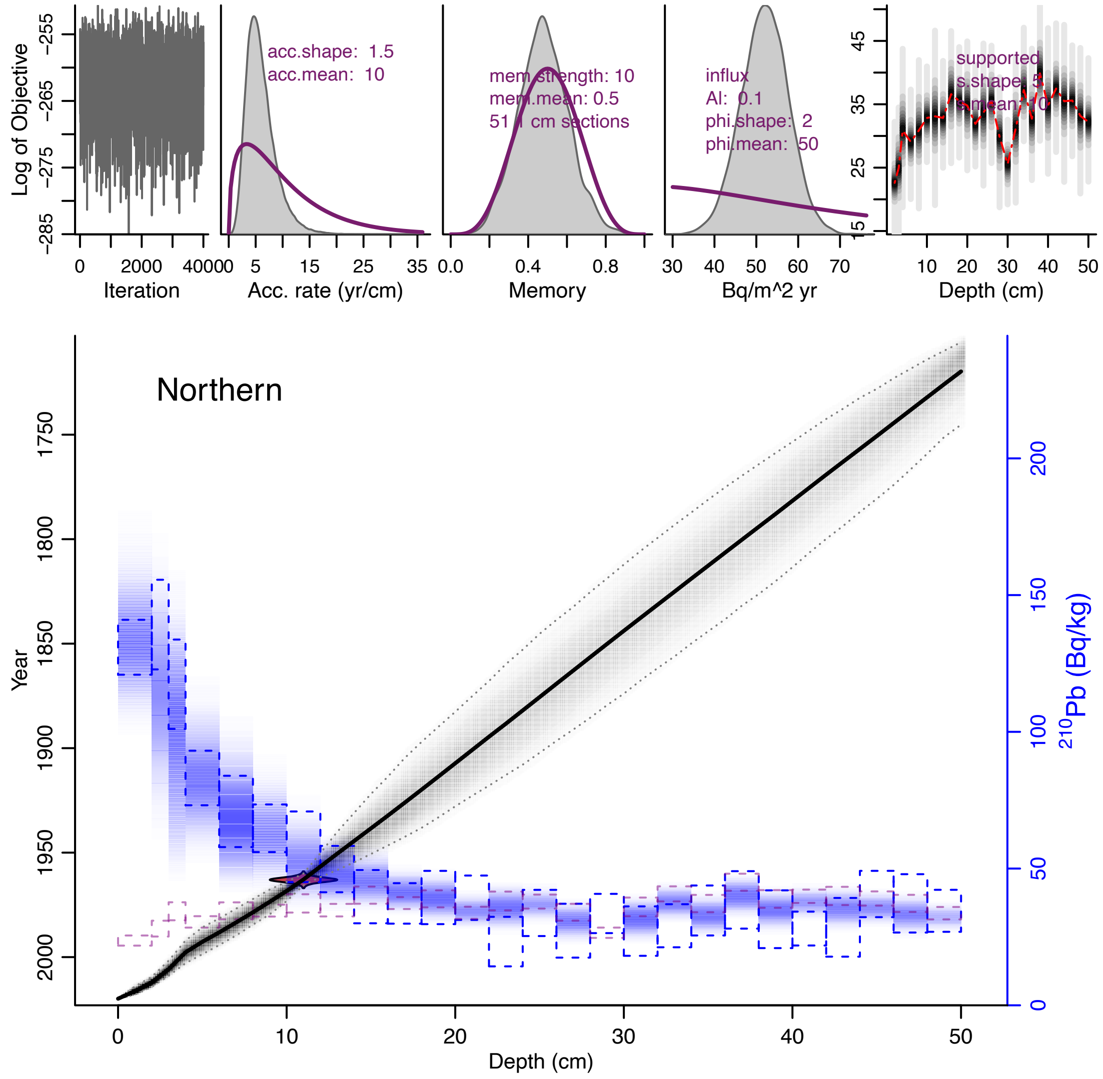

Fig. S1: MCMC chronological modeling results for the northern core. Model convergence, accretion rate, memory, $^{210}Pb_{ex}$ influx, and ^{210}Pb supported are shown in the upper row, with the purple and grey density plots showing the prior and posterior distributions, respectively. Observed ^{210}Pb (blue dashed boxes), supported ^{210}Pb (red dashed boxes), MCMC modelled ^{210}Pb (blue accordions), and the ^{137}Cs peak (red violin) are shown in the lower subplot. The resulting chronological model is indicated with the thick black line, with 95% confidence intervals illustrated by thin dashed lines.

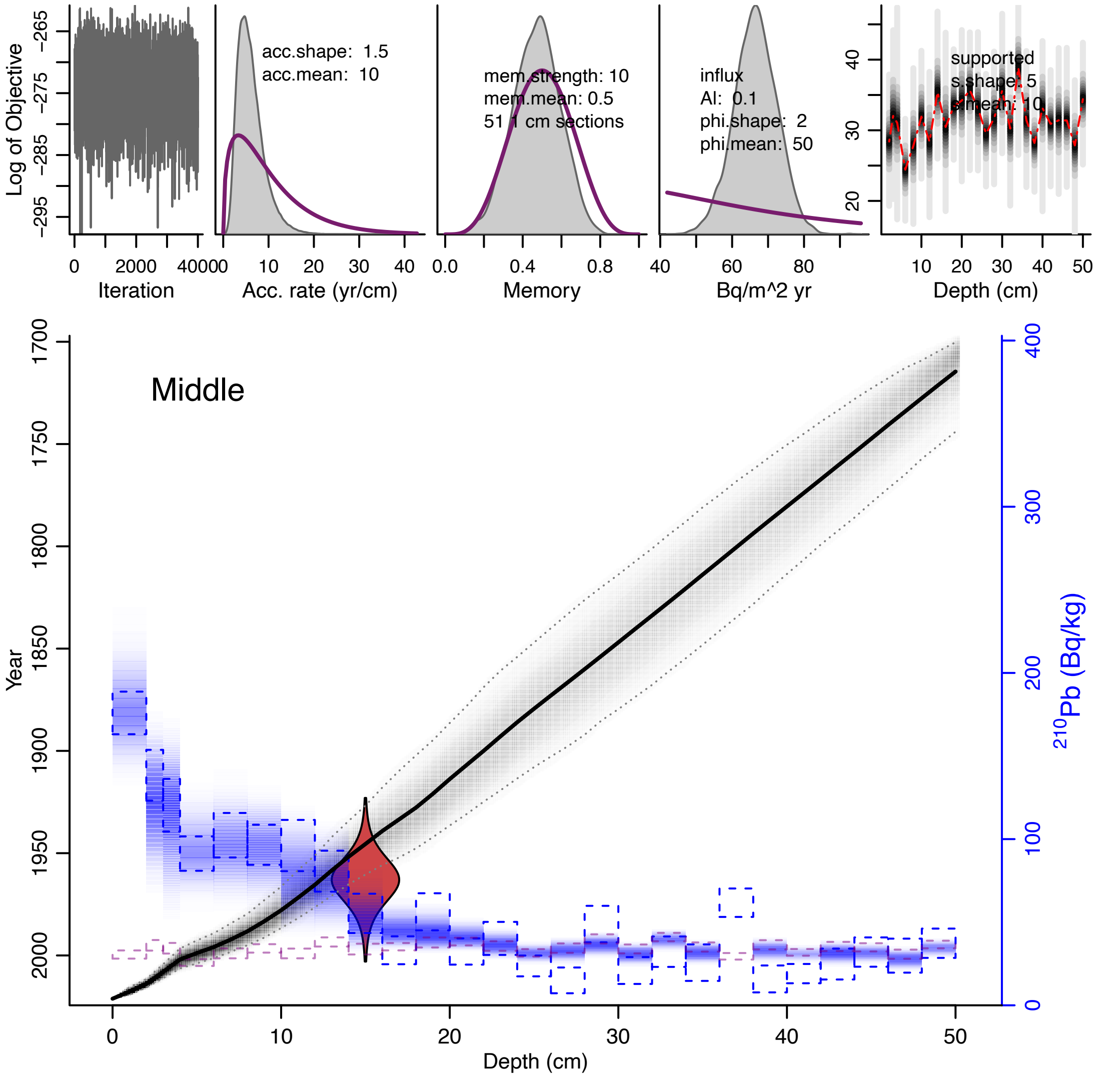

Fig. S2: MCMC chronological modeling results for the middle core. Model convergence, accretion rate, memory, $^{210}Pb_{ex}$ influx, and ^{210}Pb supported are shown in the upper row, with the purple and grey density plots showing the prior and posterior distributions, respectively. Observed ^{210}Pb (blue dashed boxes), supported ^{210}Pb (red dashed boxes), MCMC modelled ^{210}Pb (blue accordions), and the ^{137}Cs peak (red violin) are shown in the lower subplot. The resulting chronological model is indicated with the thick black line, with 95% confidence intervals illustrated by thin dashed lines.

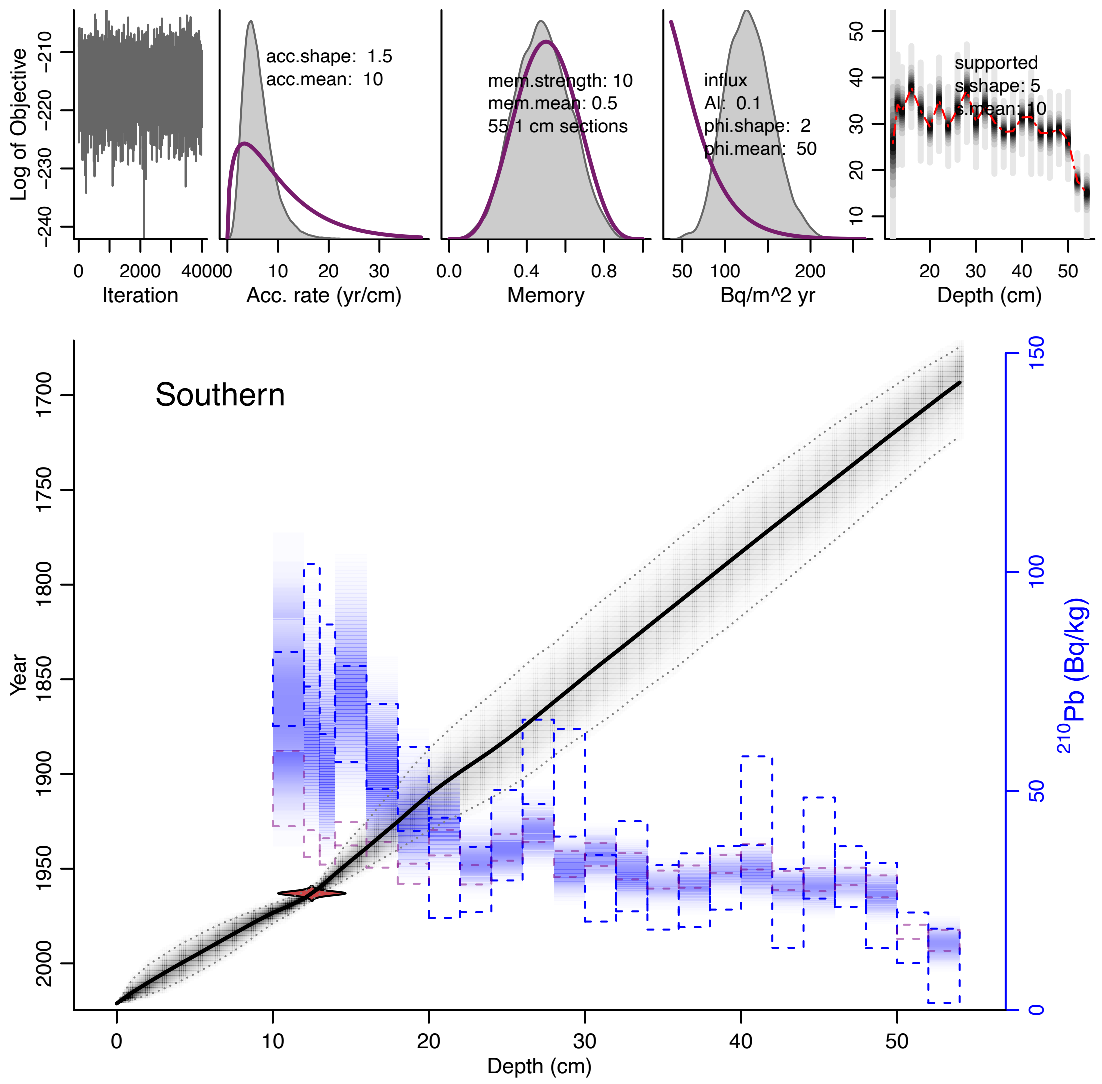

Fig. S3: MCMC chronological modeling results for the southern core. Model convergence, accretion rate, memory, $^{210}Pb_{ex}$ influx, and ^{210}Pb supported are shown in the upper row, with the purple and grey density plots showing the prior and posterior distributions, respectively. Observed ^{210}Pb (blue dashed boxes), supported ^{210}Pb (red dashed boxes), MCMC modelled ^{210}Pb (blue accordions), and the ^{137}Cs peak (red violin) are shown in the lower subplot. The resulting chronological model is indicated with the thick black line, with 95% confidence intervals illustrated by thin dashed lines. Note that 10cm of truncation was assumed at the top of the southern core.

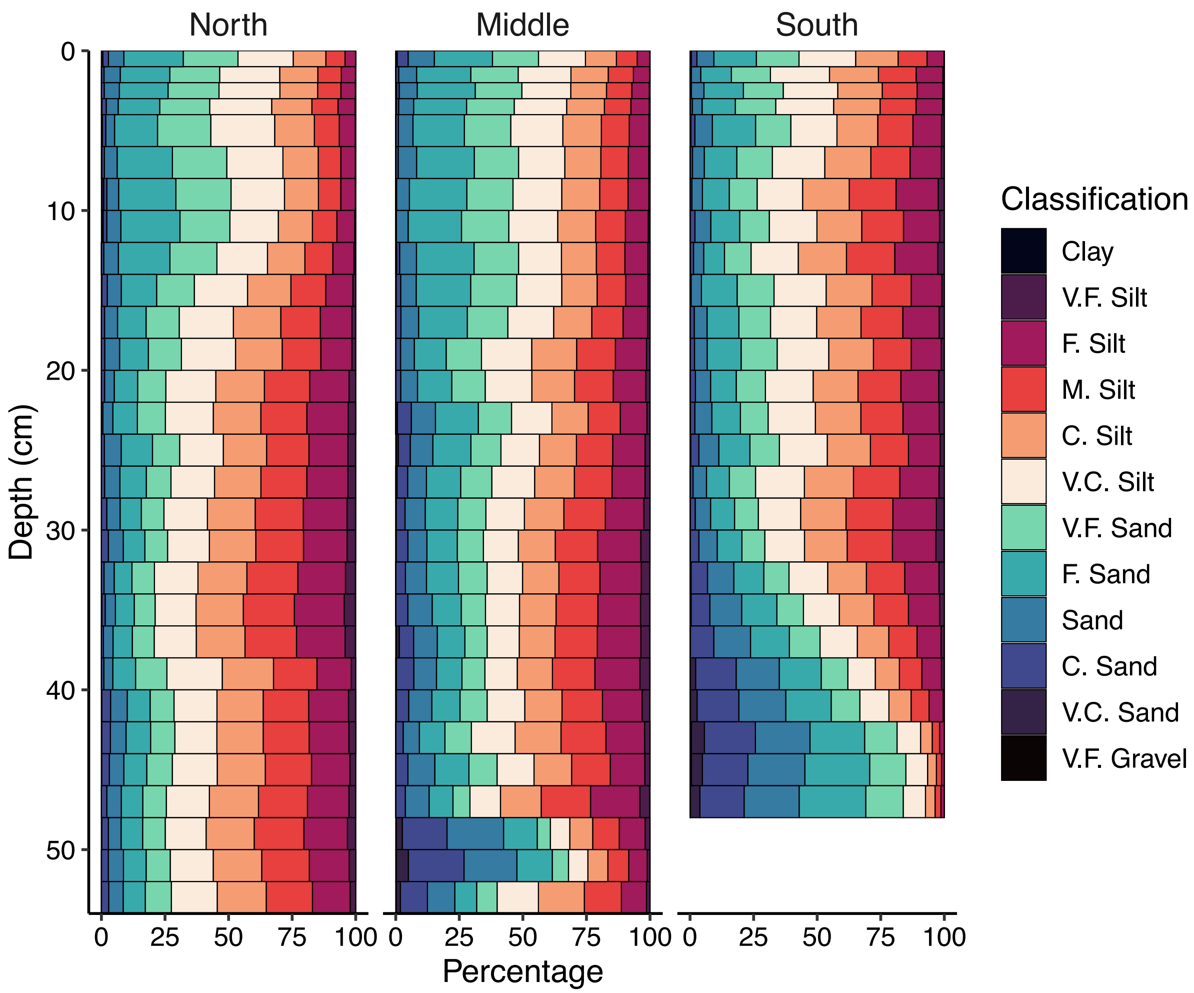

Fig. S4: Distribution of grainsize classifications with depth using definitions from Blott & Pye (2001).

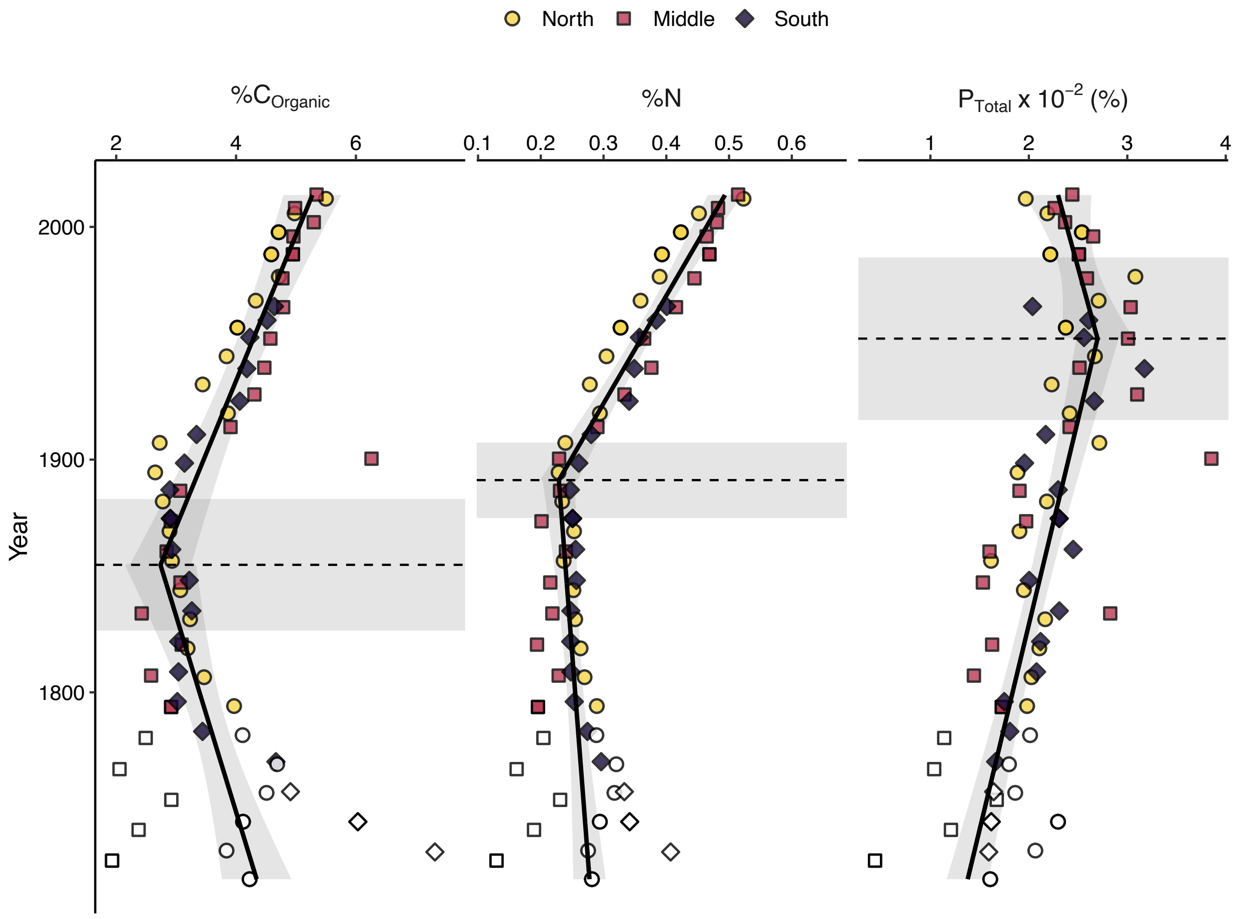

Fig. S5: Elemental composition of sediment cores. Core locations are shown by color and shape, with samples below the minimum detectable $^{210}Pb_{ex}$ activity indicated by lack of color. The solid black line shows the segmented linear regression, with changepoints and their associated 95% confidence intervals indicated with horizontal dashed lines and grey boxes, respectively.
